# Long Noncoding RNA and Circular RNA Expression Profiles of Monocyte-Derived Dendritic Cells in Autoimmune Hepatitis

**DOI:** 10.3389/fphar.2021.792138

**Published:** 2021-12-06

**Authors:** Fan Yang, Xiaoli Fan, Yifeng Liu, Yi Shen, Shenglan Zhao, Yanyi Zheng, Ruoting Men, Yan Xie, Li Yang

**Affiliations:** Sichuan University-University of Oxford Huaxi Joint Centre for Gastrointestinal Cancer, Department of Gastroenterology and Hepatology, West China Hospital, Sichuan University, Chengdu, China

**Keywords:** monocyte-derived dendritic cells (MoDCs), autoimmune hepatitis (AIH), long noncoding RNA (IncRNA), circular RNA (circRNA), microRNA, mRNA

## Abstract

Autoimmune hepatitis (AIH) is a chronic liver disease caused by disruption of liver immune homeostasis. The effect of dendritic cells (DCs) on the pathogenesis of AIH is not fully understood. Long noncoding RNAs (lncRNAs), circular RNAs (circRNAs), and microRNAs (miRNAs) have been shown to play critical roles in the regulation of cell function. In this study, we analyzed the immunophenotypic characteristics of DCs in the peripheral blood. The percentage of mature DCs was higher in AIH patients than in healthy controls (HCs), and the proportion of mature DCs decreased after treatment. We isolated monocyte-derived DCs (moDCs) from the peripheral blood, obtained whole RNA-sequencing (RNA-seq) data for the moDCs from the two groups, and identified differentially expressed (DE) lncRNAs, circRNAs, miRNAs and mRNAs. In addition, we performed Gene Ontology (GO) and Kyoto Encyclopedia of Genes and Genomes (KEGG) pathway analyses for the DE mRNAs and constructed competing endogenous RNA (ceRNA) networks. ENST00000543334, hsa_circ_0000279, and hsa_circ_0005076 were selected and validated by RT-qPCR. These results provide a possible molecular mechanism of DCs in the pathogenesis of AIH and identify some potential therapeutic targets.

## Introduction

Autoimmune hepatitis is an autoimmune liver disease characterized by antibody production, hypergammaglobulinemia, and a typical histology (Sebode et al., 2018). The precise etiology of AIH is mainly related to genetic factors, environmental factors, and immune imbalance (Manns et al., 2015). Experimental research has demonstrated that the pathogenesis of AIH might involve defects in immune tolerance, which results in T and B cell-mediated inflammation and immune reactions (Floreani et al., 2018; Webb et al., 2018).

Dendritic cells are the most efficient antigen-presenting cells in the human body and can activate naïve T cells and the immune response. DCs play important roles in autoimmune diseases (Yuan et al., 2019; Garcia-Gonzalez et al., 2016; Torres-Aguilar et al., 2010). DC dysfunction is related to overreaction of T and B cell immune responses, which leads to the breakdown of immune tolerance and production of antibodies. A series of studies have found that DCs participate in liver diseases. Das found that deletion of A20/Tnfaip3 in dendritic cells could promote autoimmune liver pathology to alter T and B-cell homeostasis (Das et al., 2019). Tan et al. (2020) demonstrated that hepatic DCs may trigger AIH *via* a deficiency in canonical Wnt/β-catenin signaling. However, the underlying mechanism of DCs in AIH is still poorly understood and remains to be elucidated.

With the recent advances in high-throughput sequencing technology, many noncoding RNAs (ncRNAs) have been identified and proven to play crucial roles in cell functions related to the pathogenesis of diseases (Cech and Steitz., 2014). These ncRNAs can be divided into microRNAs (miRNAs), long ncRNAs (lncRNAs), and circular RNAs (circRNAs) based on their length and structure (Hombach and Kretz., 2016). Increasingly, research has demonstrated that these ncRNAs can contribute to the pathogenesis of diseases by regulating the functions of DCs, including cell differentiation, migration, and metabolism (Zhou et al., 2021; Zhou and Wu, 2017; Tsai et al., 2020). For example, Wang et al. (2014) identified a lncRNA named lnc-DC that regulates DC differentiation by binding to STAT3 directly. Sun et al. (2019) found that miR-142 is central to metabolic reprogramming, specifically favoring glycolysis and the immunogenic response in DCs. In addition, Wang et al. (2018a) demonstrated that abnormal expression of lncRNAs in DCs might be related to disease activity in systemic lupus erythematosus. However, there have been few studies on ncRNAs, especially circRNAs, in AIH.

In this study, we isolated monocyte-derived DCs (moDCs) from the peripheral blood of AIH patients and HCs and generated whole RNA-sequencing (RNA-seq) data for the moDCs to identify differentially expressed (DE) lncRNAs, circRNAs, miRNAs, and mRNAs. We performed Gene Ontology (GO) and Kyoto Encyclopedia of Genes and Genomes (KEGG) pathway analyses to analyze lncRNA-miRNA-mRNA and circRNA-miRNA-mRNA interactions in DCs and constructed competing endogenous RNA (ceRNA) networks for lncRNAs, circRNAs, and mRNAs on the basis of the RNA-seq results. The combination of ncRNA expression profiles and bioinformatic analysis may provide the possible molecular mechanism of DCs in the pathogenesis of autoimmune hepatitis and indicate some potential therapeutic targets.

## Materials and Methods

### Patients and Healthy Volunteers

In this study, a total of 44 peripheral blood samples were collected from patients with AIH at the West China Hospital of Sichuan University. The diagnostic criteria adhered to the International Autoimmune Hepatitis Group (1999) guidelines (Alvarez et al., 1999; Yeoman et al., 2009). The clinical characteristics of these patients are listed in Table 1. The histological features of patients are shown in Supplementary Figure S1 and Supplementary Table S1. 36 healthy subjects were studied as controls. Among the samples, the peripheral blood samples from 27 patients and 21 normal subjects were used to analyze the proportion of mature DCs in the peripheral blood by flow analysis, and blood samples from 17 patients and 15 HCs were used to isolate moDCs for the experiments described below. The study was approved by the Independent Ethics Committee of West China Hospital and conducted in accordance with the relevant principles.

**TABLE 1:** Clinical characteristics of patients with AIH in the study.

Clinical characteristics	Amount (*n* = 44)
Age, years	51.6 ± 11.3
Female	41/44 (93.2%)
Liver function indexes	
TBil, μmol/L	87.8 ± 90.8
ALT, IU/L	214.4 ± 185.7
AST, IU/L	323.0 ± 380.3
Immunoglobulin	
IgG, IU/L	34.9 ± 48.6
ANA (+, N%)	42/44 (95.5%)

TBil, total bilirubin; ALT, alanine aminotransferase; AST, aspartate aminotransferase; IgG, immunoglobulin G; ANA, antinuclear antibody.

### Cell Culture

Differentiation of human monocyte into moDCs was performed as previously described (Wang et al., 2016). Human lymphocyte separation medium (Dakewei, Shenzhen, China) was used to isolate peripheral blood mononuclear cells (PBMCs) from whole blood samples collected from AIH patients and HCs. CD14^+^ monocytes were separated from PBMCs using CD14^+^ magnetic beads (Miltenyi Biotec, Gladbach, Germany). After magnetic bead sorting, the proportion of CD14^+^ cells were as high as 94% (Supplementary Figures S2A,B). Sorted cells were then cultured for 5–7 days in medium containing 50 ng/ml granulocyte/macrophage colony-stimulating factor (GM-CSF; Novoprotein, Shanghai, China) and 20 ng/ml IL-4 (Novoprotein, Shanghai, China) at a concentration of 1.5 × 10^6^ cells/ml. Then, 0.5 μg/ml lipopolysaccharide (LPS; Sigma–Aldrich, United States) was added to the medium on day 5, and the cells were cultured for another 1–2 days to become mature moDCs. Flow cytometry was used to identify moDCs, and the moDCs displayed high expression of CD11c (APC, BioLegend, United States) (Supplementary Figures S2C,D).

### Flow Cytometric Analysis

PBMCs collected from the peripheral blood of volunteers and moDCs obtained after 24 h of LPS stimulation were washed twice with phosphate-buffered saline (PBS, Servicebio, Wuhan, China). Then, approximately 1 × 10^6^ prepared cells were suspended in 100 μl of PBS and stained with different kinds of fluorochrome-coupled antibodies for 30 min at 4°C. Data were acquired using a CytoFLEX flow cytometer (Beckman Coulter, Life Science, United States) or NovoCyte (ACEA, Hangzhou, China), and the percentages of Lineage (CD3/14/16/19/20/56; BioLegend, United States), CD11c^+^(APC, BioLegend, United States), CD80^+^ (FITC, BioLegend, United States), CD86^+^ (PerCP-Cy5.5, BioLegend, United States) and HLA-DR^+^ (PE, BioLegend, United States) DCs were analyzed. And all these antibody isotypes including APC-IgG1, PerCP-cy5.5-IgG2b, FITC-IgG1, PE-IgG2a and FITC-IgG2b (BioLegend, United States) were used in the analysis (Supplementary Figure S3).

### Enzyme-Linked Immunosorbent Assay

Serum from AIH patients and healthy controls was collected by centrifugation of whole peripheral blood at 1,000 g for 10 min. The levels of IFN-γ, TNF-α and IL-12 were measured using human ELISA detection kits according to the manufacturer’s recommendations (MultiSciences, Hangzhou, China). The final density values were measured at 450 and 570 nm by a microplate reader (BioTek, Winooski, VT, United States).

### RNA Extraction and Quality Control

Total RNA was extracted from moDCs isolated from patients (called group A) or HCs (called group H) using TRIzol reagent (Tiangen, Beijing). An Agilent 2100 Bioanalyzer (Agilent Technologies, Santa Clara, CA, United States) and Qubit Fluorometer (Invitrogen) were used to assess RNA quality. Samples with an RNA integrity number (RIN) > 7.0 and a 28S:18S ratio > 1.8 were used in subsequent experiments.

### Library Construction and Sequencing

A total amount of 3 µg of total RNA per sample was used for the construction of a small RNA library. Following the manufacturer’s recommendations, sequencing libraries were prepared by using an Illumina TruSeq stranded total RNA with Ribo-Zero gold kit, and index codes were added to attribute sequences to each sample. The libraries were sequenced on a HiSeq X Ten platform (Illumina, San Diego, CA, United States). Similarly, a miRNA library was constructed from samples with Illumina’s TruSeq small RNA library preparation kit. The libraries were sequenced on an Illumina HiSeq 2500 sequencing platform. The whole procedures were performed by CapitalBio Technology (Beijing, China).

### Analysis of DE mRNAs, DE miRNAs, DE lncRNAs, and DE circRNAs

A paired *t* test and the Benjamini-Hochberg method were used to detect DE mRNAs and DE ncRNAs in group A versus group H. The thresholds for significantly DE mRNAs, DE miRNAs, DE lncRNAs and DE circRNAs were |log2FC|≥1 and *p* < 0.05. Novel lncRNA, named MERGE, is an assembled transcript, which removes transcripts that are smaller than 200 bp and predicted by the retention software CPC, CNCI and HMMER that there is no coding ability. miRDeep2 is used for the prediction of novel miRNAs. circRNA identification uses STAR for fusion comparison, CIRCexplored2 for back-splicing comparison, and finally determines as circRNA. Heatmaps and volcano plots were drawn to describe the DE mRNAs and DE ncRNAs.

### Coexpression Analysis of DE ncRNA-DE mRNA Pairs

Based on the raw data for DE lncRNAs, DE circRNAs, and DE mRNAs, we calculated the Pearson correlation coefficients of each DE mRNA and DE lncRNA/circRNA. Significant mRNA-lncRNA pairs and mRNA-circRNA pairs were detected by a correlation test with the thresholds of *p* < 0.05 and r < −0.8. The mRNA-lncRNA/circRNA coexpression relationships were then defined.

### Gene Function Analysis

GO analysis is a functional analysis related to DE mRNAs based on the GO categories (http://www.geneontology.org). The GO categories consist of three structured networks of defined terms named cellular component (CC), molecular function (MF), and biological process (BP). Based on the latest KEGG (https://www.genome.jp/kegg) database, KEGG pathway analysis was used to detect the biological pathways related to DE mRNAs.

### ceRNA Network Construction

ceRNA networks are used as decoys for miRNA binding, which can counteract the gene silencing activity of a miRNA. Based on the principles for ceRNA network construction, we integrated the coexpression relationships of lncRNAs and mRNAs and those of miRNAs and circRNAs and focused more on the mRNAs regulated by miRNAs and lncRNAs with a positive relationship. The difference with fold change (FC) ≥ 2 or ≤ 0.05 and *p* < 0.05 was considered statistically significant and the FDR was calculated to correct the *p* value on RNA-sequencing analysis. Besides there are other additional conditions we used for screening: the average expression level of at least one group in AIH/HC is more than 0.5; the number of expressed samples accounts for 2/3 of the total number of samples. Cytoscape was used to construct the lncRNA-miRNA-mRNA ceRNA networks. Similarly, we detected the miRNAs that could simultaneously regulate circRNAs and mRNAs and analyzed the coexpression relationships between these circRNAs and mRNAs. The circRNA-miRNA-mRNA ceRNA networks were constructed with Cytoscape.

### Quantitative Real-Time PCR

Quantitative RT-qPCR was used to validate the different expression levels of ncRNAs in moDCs from AIH patients and healthy controls. Total RNA was extracted from moDCs using TRIzol reagent (Ambion, Thermo Fisher Scientific, United States). cDNA was synthesized from 1 μg of extracted total RNA with the PrimeScrip RT reagent kit (Takara, Shiga, Japan) and amplified by real-time qPCR with SYBR Green Supermix on a CFX96 RT-qPCR detection system (BioRad, Hercules, CA, United States). The expression levels of ncRNAs were normalized to actin expression level. All primers were obtained from Tsingke (Beijing, China).

### Statistical Analysis

Statistical analysis was performed with SPSS 22.0 software and GraphPad Prims 9. A two-tailed Student’s t-test was used to detect significant differences between groups. The results are expressed as the mean ± SD, and significance was defined at *p* < 0.05. The expression level of each ncRNA was represented as 2^−Δct^ values normalized to actin expression on real-time qPCR analysis. *p* values < 0.05 were considered statistically significant.

## Results

### Circulating DCs and Cytokines in the Peripheral Blood Between HCs and AIH

**FIGURE 1:**
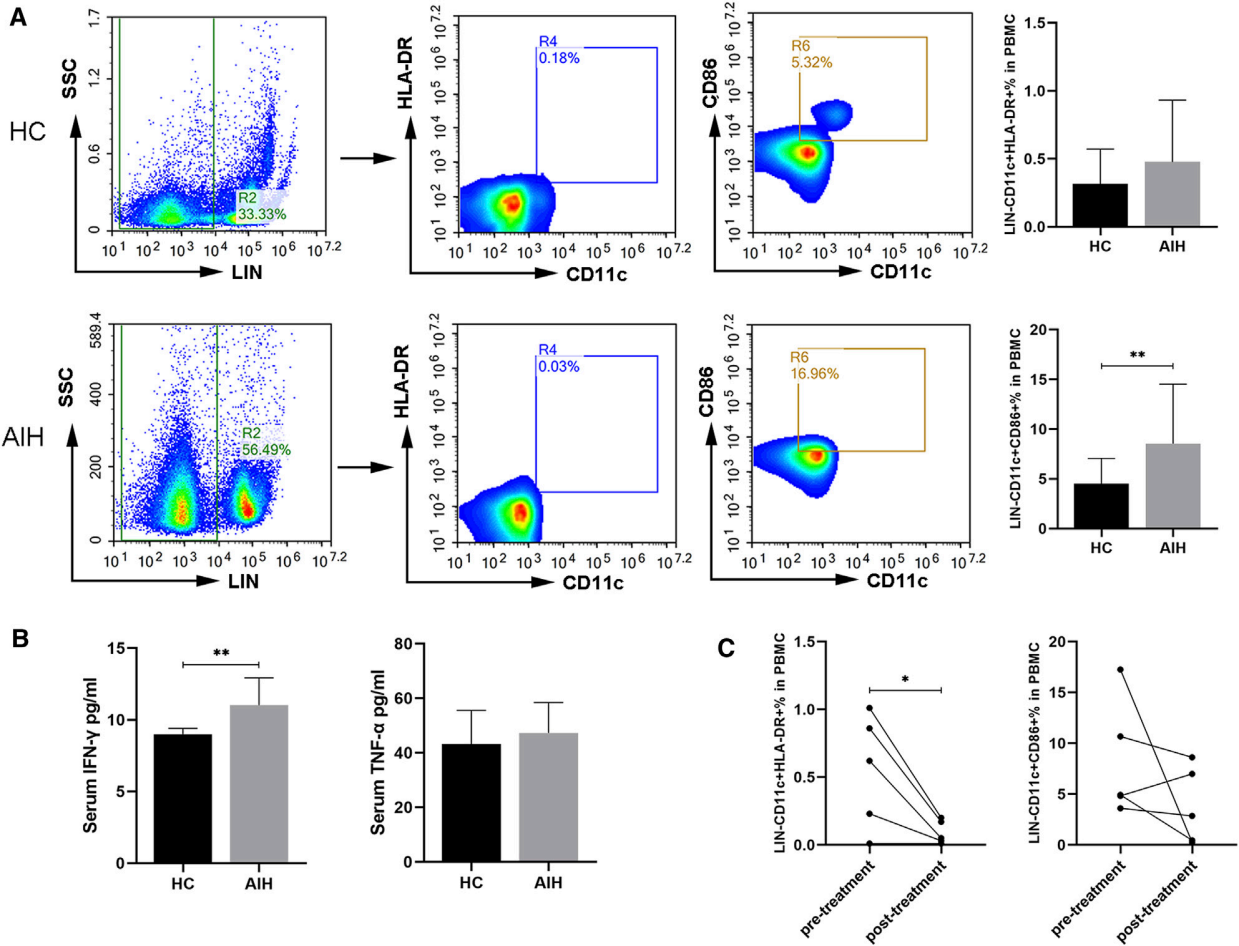
Circulating mature DCs and cytokines in the peripheral blood compared between HCs and AIH patients. **(A)** Flow cytometric analysis of the percentages of CD11c^+^CD86^+^ and CD11c^+^HLA-DR^+^ DCs between HCs and AIH patients. **(B)** Serum cytokines related to DC maturation in the peripheral blood of HCs and AIH patients. **(C)** Changes in the proportions of CD11c^+^CD86^+^ and CD11c^+^HLA-DR^+^ DCs from AIH patients before and after treatment. *p < 0.05 and **p < 0.01.

Blood samples from 27 AIH patients and 21 HCs were studied and the clinical characteristics of these patients are listed in Supplementary Table S2. We analyzed the subtypes of DCs in the peripheral blood of the patients and HCs. The percentages of CD11c^+^, CD86^+^ and HLA-DR^+^ DCs between the two groups were compared. The frequency of CD11c^+^CD86^+^ DCs was significantly higher in the patients with AIH than in the HCs. The proportion of CD11c^+^HLA-DR^+^ DCs exhibited an upward trend in the AIH patient group but without statistical significance (Figure 1A). To validate the important role of mature DCs, we performed mature DC-related cytokine analysis to assess human IFN-γ and TNF-α in the peripheral blood of patients with AIH and HCs. The levels of IFN-γ were significantly increased in the AIH group compared to the HC group, while there was no significant difference in TNF-α between the two groups (Figure 1B). Overall, circulating mature DCs may be involved in the pathogenesis of AIH. We randomly selected five patients with AIH and analyzed the proportion of mature DCs in the peripheral blood again after 2–3 weeks of meprednisone (MP) treatment. The clinical characteristics of these patients are listed in Supplementary Table S3. The level of IgG in the five patients decreased after treatment (*p* = 0.03), and total bilirubin (TBil), ALT, and AST levels showed downward trends but without significant differences (Supplementary Figure S4). In addition, we found that the proportion of mature DCs in the peripheral blood of AIH patients after treatment was lower than that before treatment. The frequency of CD11c^+^HLA-DR^+^ DCs was significantly lower after treatment than before treatment. Furthermore, the percentage of CD11c^+^CD86^+^ DCs also exhibited a downward trend but without statistical significance (Figure 1C). These results indicated that DCs participate in regulating the occurrence and development of AIH in patients.

### Differences in the Phenotype and Cytokines of moDCs From HCs and AIH Patients After LPS Treatment

**FIGURE 2:**
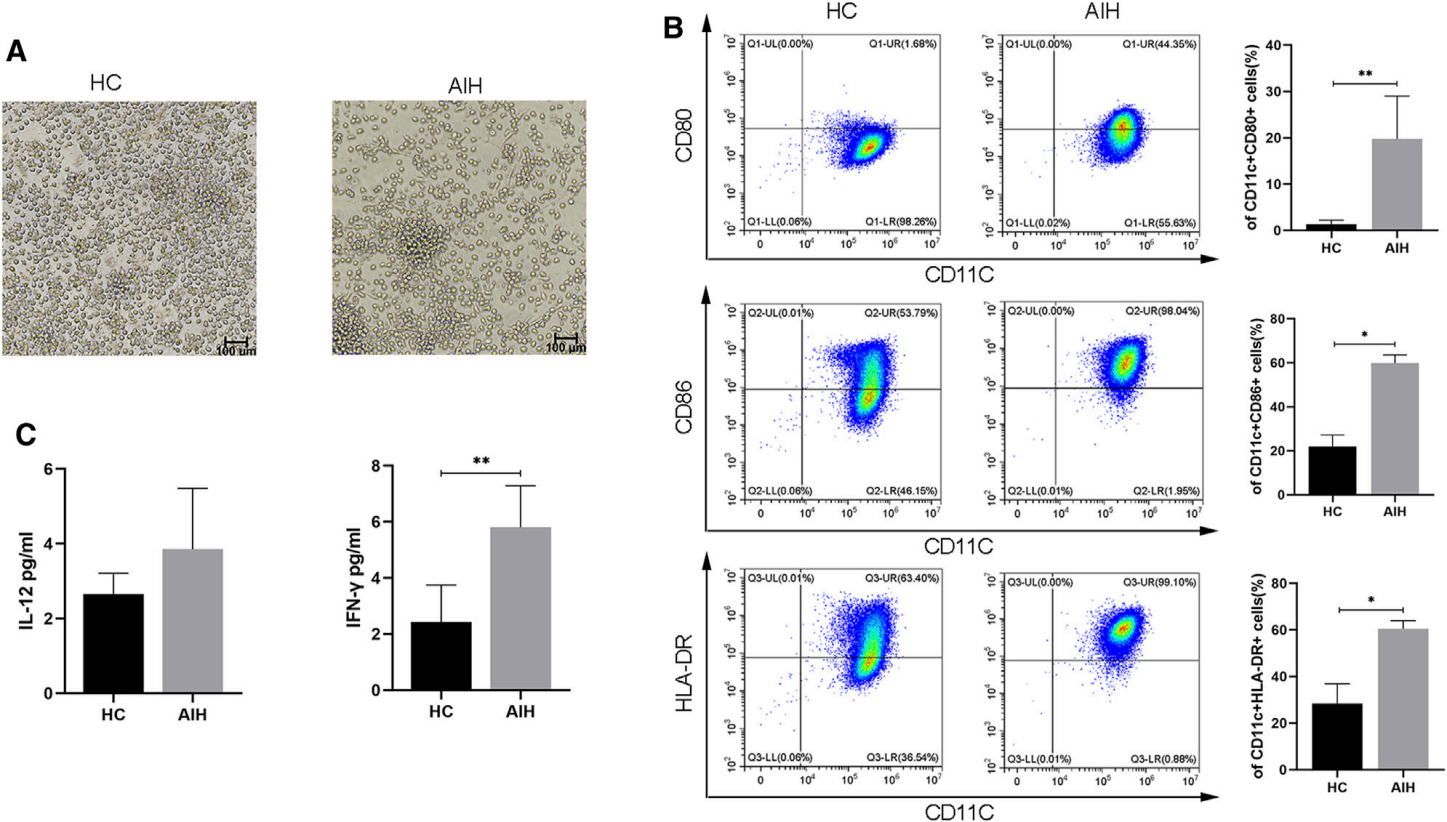
Differences in the phenotype and cytokines of moDCs from HCs and AIH patients after LPS stimulation. **(A)** Morphology of mature moDCs; magnification, 200×. **(B)** Flow cytometry was used to detect the expression of CD80, CD86 and HLA-DR between HCs and AIH patients. **(C)** The levels of the cytokines IL-12 and IFN-γ in the supernatant of cell cultures. *p < 0.05 and **p < 0.01.

We isolated moDCs from the peripheral blood of AIH patients and HCs and used GM-CSF, IL-4 and LPS to induce mature moDCs. We evaluated the morphology, immunophenotypic characteristics, and cytokines of mature moDCs from AIH patients compared with those of mature moDCs from HCs. As shown in Figure 2A, the AIH patients had more mature DCs. The mean percentage of CD11c^+^CD80^+^ DCs in the AIH patient group was significantly higher than that in the HC group (1.37 ± 0.75% vs. 19.80 ± 8.42%, *p* < 0.01). The mean proportion of CD11c^+^CD86^+^ DCs in the HC group was 21.93 ± 4.60%, which was lower (*p* < 0.05) than that in the AIH group (59.92 ± 3.40%). The mean percentage of CD11c^+^HLA-DR^+^ DCs in the AIH group was 60.51 ± 3.17%, which was higher (*p* < 0.05) than that in the HC group (28.4 ± 7.34%) (Figure 2B). We compared the supernatant levels of cytokines related to mature DCs between the AIH group and HC group. The levels of IFN-γ were significantly increased in AIH patients. The levels of IL-12 exhibited an upward trend but without statistical significance (Figure 2C).

### Differential Expression Patterns of lncRNAs, circRNAs, miRNAs, and mRNAs

**FIGURE 3:**
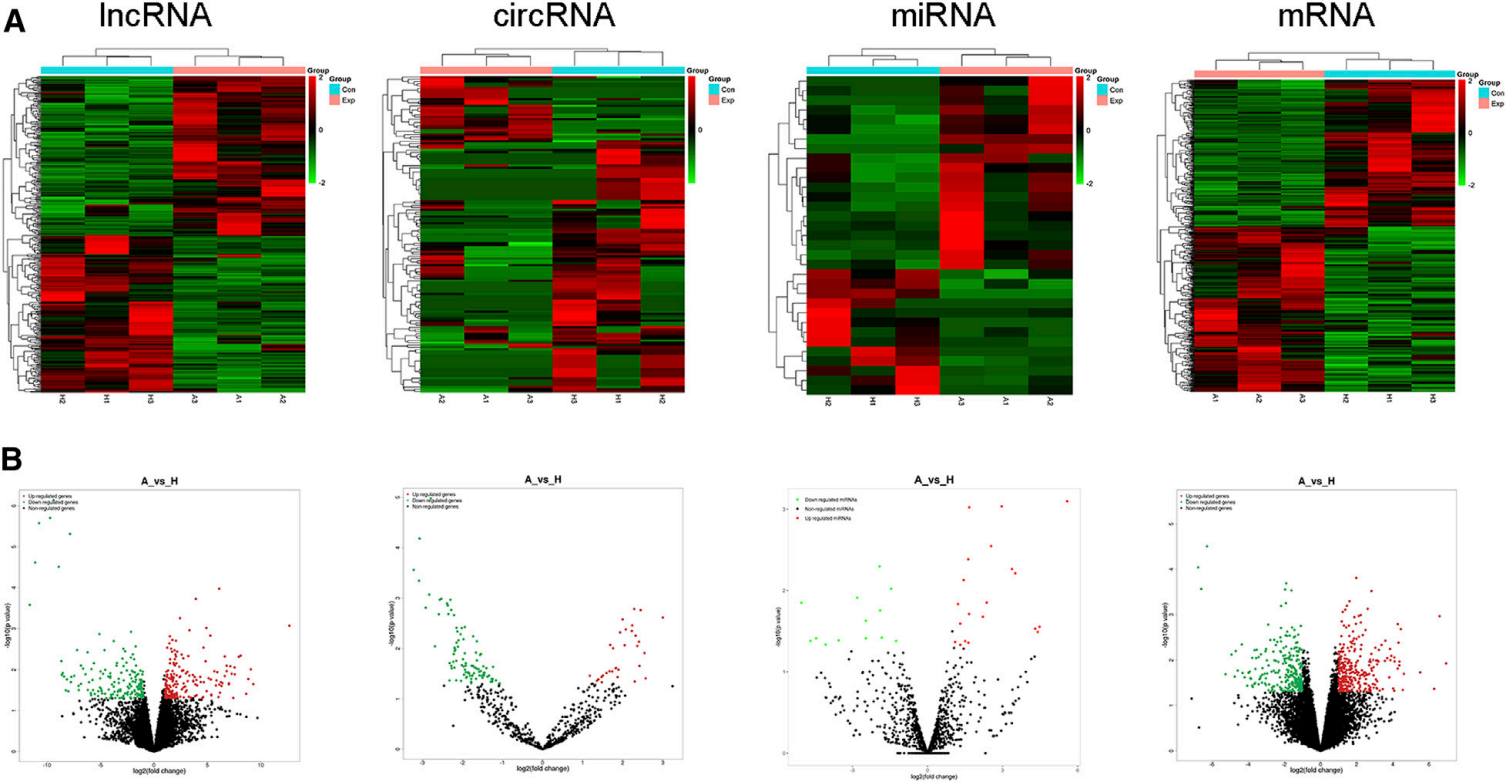
Expression profiles of lncRNAs, circRNAs, miRNAs, and mRNAs. Differently expressed ncRNAs and mRNAs in the HC group and AIH group. **(A)** Volcano plot. **(B)** heatmap.

According to whole RNA-seq data, we analyzed DE ncRNAs (lncRNAs, circRNAs, miRNAs) and mRNAs with |log2FC| ≥ 1 and *p* < 0.05. The DE ncRNAs identified between the two groups are shown in a heatmap (Figure 3A) and volcano plot (Figure 3B). In total, there were 352 DE lncRNAs (178 upregulated and 174 downregulated), 143 DE circRNAs (32 upregulated and 111 downregulated), 33 DE miRNAs (20 upregulated and 13 downregulated), and 550 DE mRNAs (292 upregulated and 258 downregulated) in the moDCs of patients with AIH compared to those of HCs. In addition, the most upregulated lncRNA, circRNA, miRNA and mRNA were MERGE.24561.12, hsa_circ_0000279, hsa-miR-7844-5p, and AP000295.1, respectively. The most downregulated lncRNA, circRNA, miRNA and mRNA were MERGE.3901.1, hsa_circ_0006719, hsa-miR-889-3p, and ST13P19, respectively (Table 2).

**TABLE 2:** Statistical analysis of all differently expressed ncRNAs and mRNAs.

DE RNAs	Total no.	No. of upregulated	No. of downregulated	Most upregulated	Most downregulated
lncRNAs	352	178	174	MERGE.24561.12	MERGE.9467.24
circRNAs	143	32	111	hsa_circ_0000279	hsa_circ_0006719
miRNAs	33	20	13	hsa-miR-7844-5p	hsa-miR-889-3p
mRNAs	550	292	258	AP000295.1	ST13P19

MERGE, novel lncRNA, an assembled transcript.

### Bioinformatic Analysis and Constructed ceRNA Networks of DE ncRNAs and mRNAs

**FIGURE 4:**
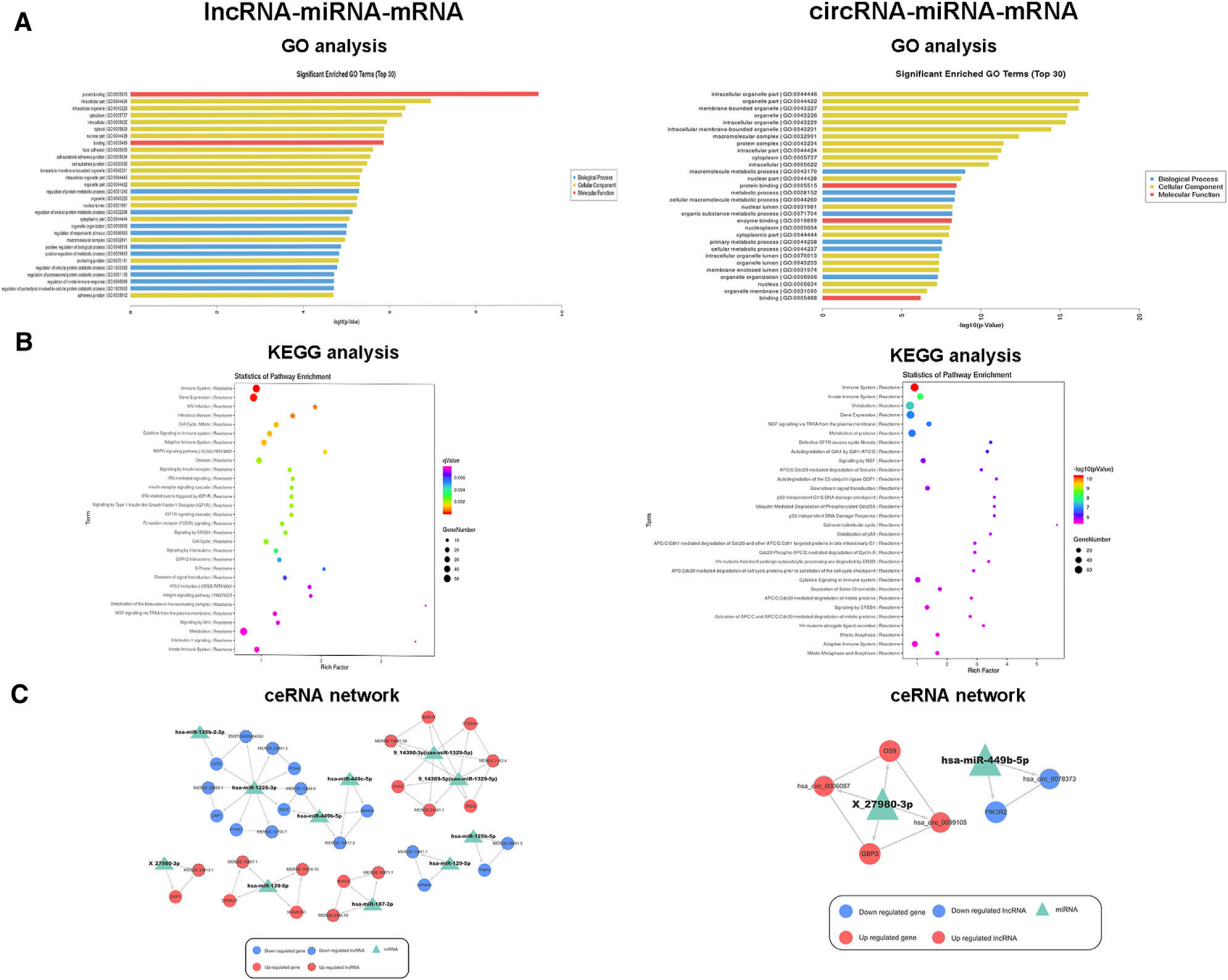
Functional analysis and ceRNA network of DE ncRNAs and mRNAs. **(A)** Top 30 terms from a GO analysis of DE mRNAs related to lncRNA-miRNA and circRNA-miRNA pairs. **(B)** KEGG pathway analysis of DE mRNAs and the top 30 pathways. **(C)** lncRNA-miRNA-mRNA and circRNA-miRNA-mRNA ceRNA networks. The triangle indicates a miRNA, the circle represents an mRNA, the circle and the outer gray circle represent a lncRNA or circRNA, red and blue indicate upregulation and downregulation, respectively, and green indicates unknown.

GO analysis was conducted to identify the mRNAs involved in lncRNA-miRNA-mRNA and circRNA-miRNA-mRNA networks. As shown in Figure 4A, GO analysis divided these DE mRNAs into three terms: biological process, cellular component, and molecular function. In the lncRNA-miRNA-mRNA network, the DE mRNAs were mainly involved in protein binding (molecular function), the intracellular part (cellular component), and the regulation of a protein metabolic process (biological process). In the circRNA-miRNA-mRNA network, the DE mRNAs were mainly involved in the intracellular organelle part (cellular component), a macromolecule metabolic process (biological process), and protein binding (molecular function). We performed KEGG pathway analysis to detect the key pathways and relationships between the mRNAs and ncRNAs. KEGG pathway analysis revealed the top 30 pathways enriched in the DE mRNAs related to the coexpression network. In the lncRNA-miRNA-mRNA networks, signal transduction, MAPK signaling pathway, Wnt pathway and the immune system were the most enriched pathways. In the circRNA-miRNA-mRNA networks, cytokine signaling pathway, metabolism, and the immune system were the most enriched pathways (Figure 4B). Tables 3, 4 list the top 10 coexpressed pairs of lncRNAs/circRNAs and mRNAs in moDCs. Then, we combined the results of the miRNA-lncRNA/circRNA joint analysis and miRNA-mRNA joint analysis. Due to the complicated and huge ceRNA networks, we used more stringent parameters to reduce the number of networks. We illustrated the ceRNA networks based on the key DE mRNAs including CIITA, PDXK, LRP1, PTPN7, RBM39, OAS2, WDR19, IRGQ, ST8SIA4, OS9, SKP1, OXNAD1, SMARCB1, BUD23, GPNMB, TNIP2, GPB3 and PIK3R2. Then 24 lncRNA-miRNA-mRNA pathways were constructed, including 16 lncRNAs, 11 miRNAs and 16 mRNAs. Five circRNA-miRNA-mRNA pathways were constructed, including three circRNAs, two miRNAs and three mRNAs (Figure 4C).

**TABLE 3:** The top 10 coexpressed lncRNAs and mRNAs in moDCs.

lncRNA	mRNA	Correlation	*p*-value	mRNA	Fold change
MERGE.19964.8	ENST00000265758	0.999831904	4.238179E-08	BUD23	4.190155641
MERGE.9125.1	ENST00000517951	0.999703934	1.314697E-07	ADAM19	2.988814304
MERGE.8108.1	ENST00000449352	0.999386649	5.641832E-07	FAM160A2	5.38741654
ENST00000556072	ENST00000447388	0.999334139	6.649095E-07	NFYC	-2.176528834
MERGE.18517.3	ENST00000361162	0.999291708	7.523386E-07	RBM39	-2.836971855
MERGE.3440.2	ENST00000504221	0.99915166	1.079217E-06	CAST	2.110414386
MERGE.719.1	ENST00000501038	-0.999098307	1.219210E-06	PLXND1	-1.092275202
MERGE.24085.1	ENST00000472509	0.999078497	1.273359E-06	TAF6	-1.509317786
MERGE.24085.1	ENST00000459656	-0.999071158	1.293721E-06	SPATS2L	1.785198231
ENST00000421004	ENST00000472509	-0.999019135	1.442671E-06	TAF6	-1.509317786

MERGE, novel lncRNA, an assembled transcript.

**TABLE 4:** The top 10 coexpressed circRNAs and mRNAs in moDCs.

circRNA	MRNA	Correlation	*p*-value	mRNA	Fold change
CBT15_circR_2782	ENST00000556184	0.999964542	1.885840E-09	SRSF5	−5.578158076
CBT15_circR_987	ENST00000540691	-0.999961141	2.264999E-09	KXD1	−3.092575982
CBT15_circR_2628	ENST00000540691	-0.999961141	2.264999E-09	KXD1	−3.092575982
CBT15_circR_2652	ENST00000540691	-0.999961141	2.264999E-09	KXD1	−3.092575982
CBT15_circR_1706	ENST00000556184	0.999960282	2.366303E-09	SRSF5	−5.578158076
CBT15_circR_1206	ENST00000540691	-0.999922482	9.013400E-09	KXD1	−3.092575982
CBT15_circR_2061	ENST00000540691	0.999890505	1.798301E-08	KXD1	−3.092575982
CBT15_circR_2632	ENST00000556184	0.999885106	1.980009E-08	SRSF5	−5.578158076
CBT15_circR_1038	ENST00000540691	-0.999882106	2.084761E-08	KXD1	−3.092575982
CBT15_circR_1931	ENST00000556184	0.999870383	2.519985E-08	SRSF5	−5.578158076

### Validation of DE lncRNAs and circRNAs by qRT-PCR

**FIGURE 5:**
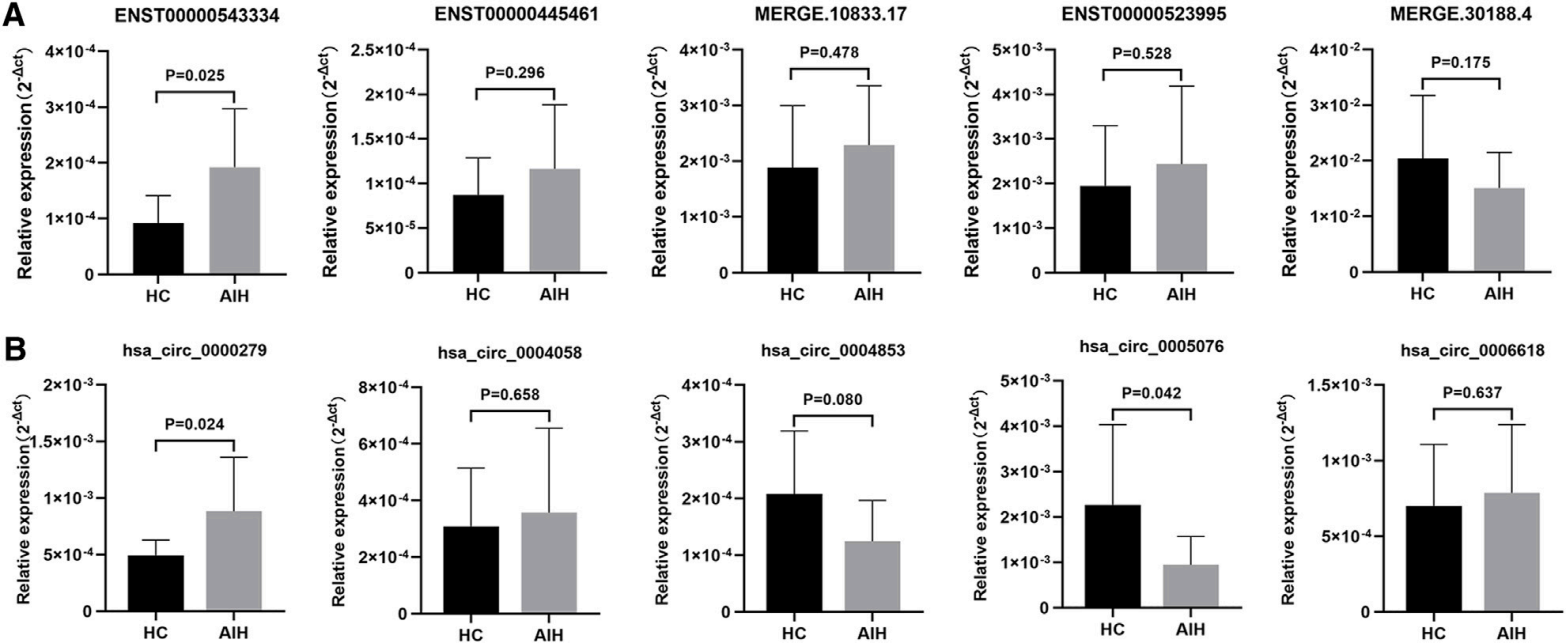
Validation of DE lncRNAs and circRNAs in moDCs from HCs and AIH patients. DE lncRNAs **(A)** and circRNAs **(B)** were confirmed by qPCR. The expression trends of candidate lncRNAs and circRNAs were consistent with the RNA-seq results. Data are presented as 2^−Δct^ values normalized to ACTIN expression (mean ± standard deviation).

To validate our RNA-seq data, we selected five DE lncRNAs and DE circRNAs respectively and enlarged the sample sizes to measure the expression levels of these ncRNAs among 17 AIH patients and 15 healthy controls by using qRT-PCR. The selection criteria of these ncRNAs were as followings: The difference with fold change (FC) ≥ 2 or ≤ 0.05 and *p* < 0.05 was considered statistically significant. Besides the average expression level of at least one group in AIH/HC is more than 0.5 and the number of expressed samples accounts for 2/3 of the total number of samples. The clinical characteristics of these patients are listed in Supplementary Table S4 and the primers we used are listed in Table 5. The experimental results indicated that the expression levels of ENST00000543334 and hsa_circ_0000279 were upregulated, while those of hsa_circ_0005076 were downregulated, which was consistent with the RNA-seq data. In addition, the expression levels of ENST00000445461, ENST00000523995, hsa_circ_0004058, and hsa_circ_0004853 showed the same trends as the RNA-seq results but without significance (Figure 5).

**TABLE 5:** The sequences of the primers used in qRT-PCR experiments.

lncRNA	Primers	circRNA	Primers
ENST00000543334	F: CCA​GCC​GTT​CGT​CTT​TAC​CT	hsa_circ_0000279	F: CCT​GAA​ATT​CTG​GCT​TGC​CA
	R: TCC​AGG​AGG​ACT​CAT​GGG​AG		R: TCC​TGT​AGC​TTG​GTC​CTT​TCA
MERGE.10833.17	F: GGA​GAT​GCA​CCT​CTC​TCA​AGT	hsa_circ_0004058	F: AAA​CCC​GGT​ACT​GTG​CAC​TA
	R: AAT​CAG​TGC​CCA​TTG​CAG​GA		R: GAG​GTA​AGA​TAA​GGT​CGG​GCT
MERGE.30188.4	F: CGACCTAAGGGAGTGATG	hsa_circ_0004853	F: TGC​GAT​CCT​TAT​GGC​TCA​T
	R: TGTAAGCGGAGAAGAATA		R: CCA​GTT​CGG​CTT​GCT​TCT​T
ENST00000445461	F: GCA​AAG​TGC​ACA​GCT​GCA​TA	hsa_circ_0005076	F: ACA​GAG​GCG​GGT​TGT​TTA​CA
	R: CCA​ACA​CCC​AAA​AGC​CCA​GA		R: CAG​TCT​CTG​GCT​GTT​CTC​GA
ENST00000523995	F: GGA​GAT​GCA​CCT​CTC​TCA​AGT	hsa_circ_0006618	F: GGA​AGT​TGT​GAT​TGC​CCA​GA
	R: AAT​CAG​TGC​CCA​TTG​CAG​GA		R: CCT​GCA​TGG​CTG​TAC​GAT​CT

MERGE, novel lncRNA, an assembled transcript. F, forward; R, reverse

## Discussion

Accumulating research have revealed that DCs are involved in autoimmune diseases, such as systemic lupus erythematosus (SLE), rheumatoid arthritis (RA) and multiple sclerosis (MS) (Price and Tarbell., 2015; Coutant and Miossec., 2016). Zheng et al. (2019) found that B7-H3 molecular on dendritic cells suppressed the production of autoantibodies and act as a potential target for the treatment of SLE. Schittenhelm et al. (2021) demonstrated that the integrin expression patterns (CD11a/CD11b) on DCs might regulate inflammation for intervention in RA. However, the underlying mechanism of DCs in AIH still remains to be elucidated. In the current study, we analyzed the phenotypic differences in DCs and related cytokines in the peripheral blood between AIH patients and HCs. MHC-II molecules (HLA-DR) and costimulatory molecules (CD80/CD86) are both crucial costimulatory molecules involved in T cell activation, which may affect the immune response (Clark et al., 2019; Carenza et al., 2019). Our data demonstrated that the percentages of CD11c^+^CD86^+^ and CD11c^+^HLA-DR^+^ DCs in AIH patients were upregulated compared with those in HCs, which was consistent with our previous study (Fan et al., 2020). This may suggest improved DC-mediated antigen presentation in patients with AIH. In addition, we found that the proportions of CD11c^+^CD86^+^ and CD11c^+^HLA-DR^+^ DCs in AIH patients decreased after treatment, which further indicated that mature DCs may be involved in the development of AIH. Then, we isolated DCs from the peripheral blood and used GM-CSF, IL-4, and LPS to induce mature DCs before investigating the phenotypic and maturation-related cytokines of these DCs. The expression of CD80, CD86, and HLA-DR was also higher in the AIH group than in the HC group. Pro-inflammatory cytokines, such as TNF-α, IFN-γ, IL-12, play an important part in the antigen presentation process of DCs to T cells (Mbongue et al., 2014; Tkach et al., 2017). These cytokines are required in increasing CD4^+^ T cells differentiation. Our results showed increased serum IFN-γ level in the AIH group and in the culture supernatant of moDCs. However, the level of IL-12 exhibited a nonsignificant upward trend, which may need further study with an expanded sample size. Our findings found that immunogenic maturation of DCs might participate in the development and pathogenesis of autoimmune hepatitis.

Increasingly, research has focused more on the potential mechanism of mRNAs and ncRNAs (miRNAs, lncRNAs, and circRNAs) involved in different kinds of human diseases, especially in autoimmune diseases (Wang et al., 2019; Long et al., 2018; Espinoza et al., 2020). Wang et al. (2018b) reported that exosomes-delivered miR-548a-3p regulated inflammatory response *via* TLR4/NF-κB signaling pathway in RA. Zheng et al. (2017) provided circRNA expression profiles and their corresponding microRNA binding sites of peripheral blood mononuclear cells in patients with RA. As the major population of RNAs, ncRNAs tend to not encode proteins but can serve as modulators by regulating gene expression in cis or trans, promoting demethylation, and controlling mRNA processing (Beermann et al., 2016). Our study selected five DE lncRNAs and DE circRNAs, respectively and validated them by qRT-PCR. Based on the results, ENST00000543334, hsa_circ_0000279, and hsa_circ_0005076 were shown to be differentially expressed, which was in accordance with the RNA-seq results. The expression levels of other ncRNAs showed the same trend as the RNA-seq results but without statistical significance, which may need an expanded sample size to confirm.

ENST00000543334 is a about 1,500 bp intergenic lncRNA transcript, also known as LINC01089/LIMT (LncRNA Inhibiting Metastasis). Several studies have proved the biological function of LINC01089 in various cancers (Li X. et al., 2020; Wang and Yang., 2020; Yang et al., 2021). Guo and Li (2020) revealed that LINC01089 could impede the proliferation, migration, and invasion of gastric cancer cells *via* miR-27a-3p/TET131 axis. Zhang et al. (2021) found that up-regulation of LINC01089 could inhibit the progression of non-small cell lung cancer as a ceRNA for miR-152-3p. Although there is no report about LINC01089 regulation in autoimmune diseases, the expression levels of ENST00000543334 in our results revealed the same trend as these studies, which showed the potential regulatory function of LIN01089 in DCs from patients with AIH. Besides, our GO and KEGG analysis showed that these DE lncRNAs and mRNAs mainly associated to MAPK signaling pathway and Wnt signaling pathway. Ye et al. (2020) revealed that lncRNA NEAT1 regulated the activation of MAPK signaling pathway to participate in the pathogenesis of Sjögren’s syndrome. Han et al. (2019) found that lncRNA PTPRE-AS1 could modulate the activation and function of M2 macrophage via MAPK pathway. And Tian et al. (2020) reported that lncRNA LINC00662 activated Wnt/β-catenin signaling to promote M2 macrophage polarization. Therefore, LINC01089 might regulate the function of DCs *via* MAPK or Wnt pathway to involve in the pathogenesis of AIH. However, the specific binding sites and mechanisms of LINC01089 requires in-depth study to find out. hsa_circ_0000279 and hsa_circ_0005076 are both circular RNAs composed of exonic sequence described in HPS5 and MRPS6. HPS5 encodes a protein related to the organelle biogenesis in melanosomes, platelet dense granules, and lysosomes. MPRS6 plays a role in protein synthesis within mitochondrion. Increasing research have reported that circRNAs are involved in autoimmune diseases (Xia et al., 2019). Zhang et al. (2018) demonstrated that GDF15 could induce tolerogenic DCs through inhibition of circ_Malat-1 and the NFκB signaling pathway to prevent alloimmune rejection in transplantation. However, there is seldom study on circRNAs in AIH. Our GO analysis indicated that protein binding, intracellular components, and protein metabolic process regulation might contribute to the immunoregulatory function of DCs. The subsequent KEGG pathway analysis showed that these DE molecules mainly related the cytokine signaling pathway and metabolic pathways. Chen et al. (2020) identified circSnx5 in regulating DC-driven immunity by acting as a miR-544 sponge on suppressor of cytokine signaling 1 (SOCS1) and inhibiting nuclear translocation of PU.1. Some study reported that circRNA might contribute to mutant glycolysis, lipogenesis, and oxidative respiration to regulate cellular metabolism (Yu et al., 2019). Therefore, we assumed that hsa_circ_0000279 and hsa_circ_0005076 might participate in the process of secreting cytokine and cellular metabolism to regulate the function of DCs in patients with AIH, which needs to be further confirmed.

Recently, ceRNA networks have been concluded to play a crucial role in regulating gene translation (Kartha and Subramanian., 2014). Normally, ceRNAs are considered to consist of different kinds of RNAs. Based on the shared miRNAs, ceRNA regulatory networks include lncRNA-miRNA-mRNA and circRNA-miRNA-mRNA axes. Increasing evidence has proven that ceRNA networks play crucial roles in the progression of diseases (Smillie et al., 2018; Yang et al., 2018; An et al., 2017). Zhang et al. (2019) identified that the lncRNA NEAT1 can act as a decoy for miR-3076-3p to induce tolerogenic DCs (tol-DCs) by shaping T-cell responses in the experimental models of autoimmune myocarditis and heart transplantation. Wu et al. (2018) also found that overexpression of the lncRNA MALTA1 promoted dendritic cell-specific intercellular adhesion molecule-3 grabbing nonintegrin (DC-SIGN) expression to induced tolerogenic DCs (tDCs) *via* the miRNA-155 sponge in experimental autoimmune myocarditis. However, there are few reports on AIH, especially regarding the ncRNAs of DCs in AIH patients. Our results list the top 10 coexpressed pairs of lncRNAs/circRNAs and mRNAs in moDCs and constructed ceRNA networks based on the coexpression of ncRNAs and mRNAs and the shared miRNAs. We simplify the ceRNA networks with stringent parameters and reconstructed the regulatory ceRNA networks including 16 lncRNAs, three circRNAs, 13 miRNAs and 19 mRNAs. Of them, two predicted pathways, lncRNA MERGE.24591.1/has-miR-1329-5p/OAS2 and has_circ_0078373/has-miR-449b-5p/PIK3R2 showed an important role in regulation. There have been several reports about OAS2 and PIK3R2 in various diseases. Gao et al. (2020) previously reported that lncRNA MALAT1 up-regulate OAS2 expression to promote the effect of IFN-α in systemic lupus erythematosus. Li et al. R. et al. (2020) revealed that the activation of PI3K/AKT/mTOR signaling pathway could induce the maturation and function of dendritic cells. However, there is no report about lncRNA MERGE.24591.1 or has_circ_0078373 related to these two pathways in autoimmune hepatitis. And our results detected that the expression levels of them showed the same trend as the RNA-seq results but without statistical significance (Supplementary Figure S5), which may need an expanded sample and further study to verify. In this study, we first reported the potential mechanism of these molecules to provide a direction for future research.

The limitation of our study is that all AIH patients and healthy controls are Chinese. Our results may not reflect to patients of other ethnic backgrounds. Another limitation is that we were unable to recruit more patients and healthy controls to investigate the functions of these DE ncRNA and target genes. Because AIH is a chronic liver disease with a very low incidence. Further studies need to carry out to understand the role of these molecules in the pathogenesis of AIH. The purpose of this study is to explore the dysregulated ncRNAs in the DCs of patients with AIH, making a preliminary discussion of the putative target genes and potential related pathways, and give some hints to future studies.

In summary, our study provided comprehensive data for the lncRNAs, circRNAs, miRNAs and mRNAs of moDCs in AIH and proved that the DE lncRNAs and circRNAs of DCs could be involved in the pathogenesis of AIH. These results indicate a direction for further study of AIH-related ncRNAs and mRNAs and provides potential therapeutic targets in AIH.

## Data Availability

The datasets presented in this study can be found in online repositories. The names of the repository/repositories and accession number(s) can be found in the article/Supplementary Material.
