# Supplementary figures and images for: Long Noncoding RNA and Circular RNA Expression Profiles of Monocyte-Derived Dendritic Cells in Autoimmune Hepatitis
Source: Front Pharmacol. 2021 Dec 6;12:792138. doi: 10.3389/fphar.2021.792138 (PMC8685411; doi:10.3389/fphar.2021.792138)

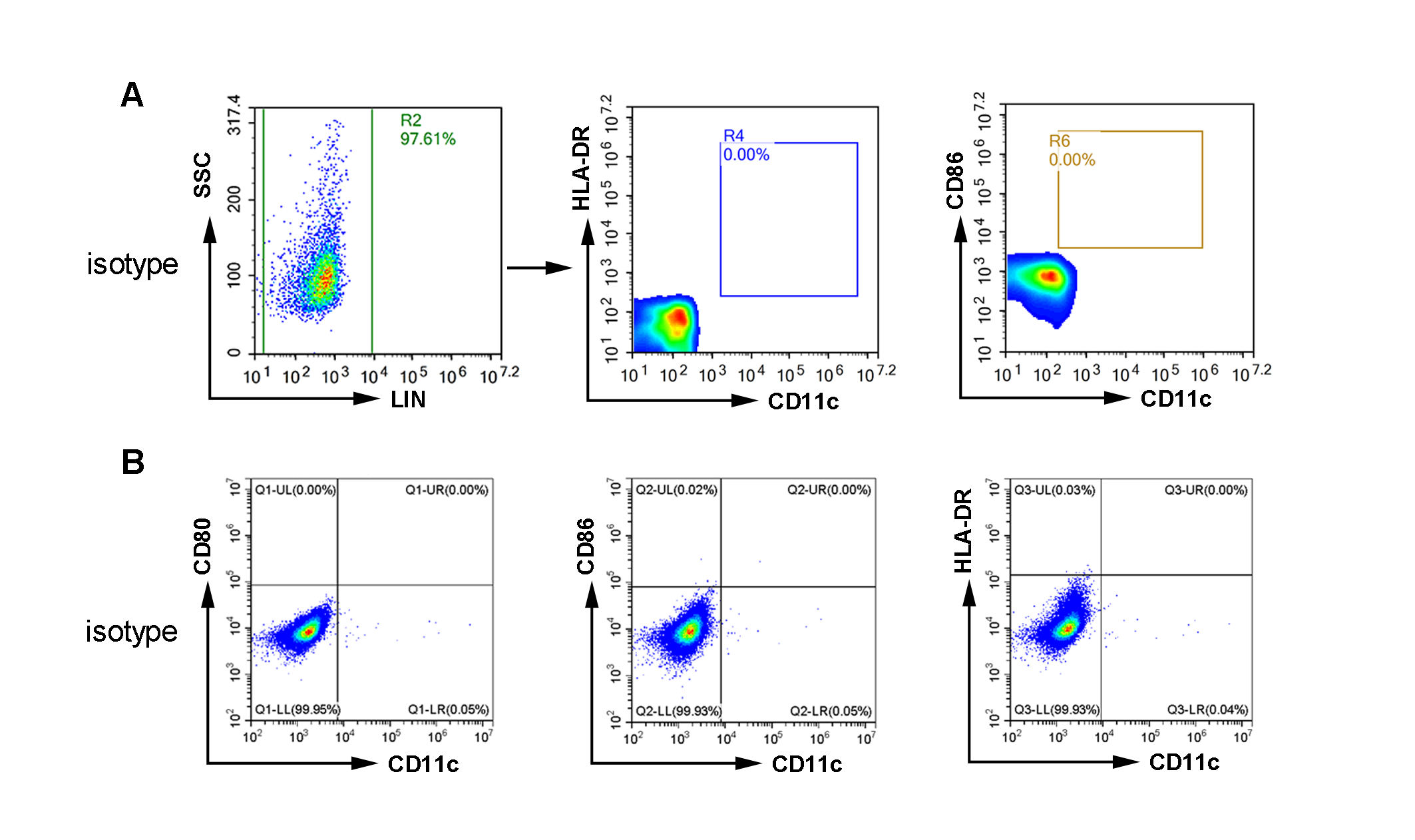

Supplement: Supplementary file 3 [file Image3.TIF]

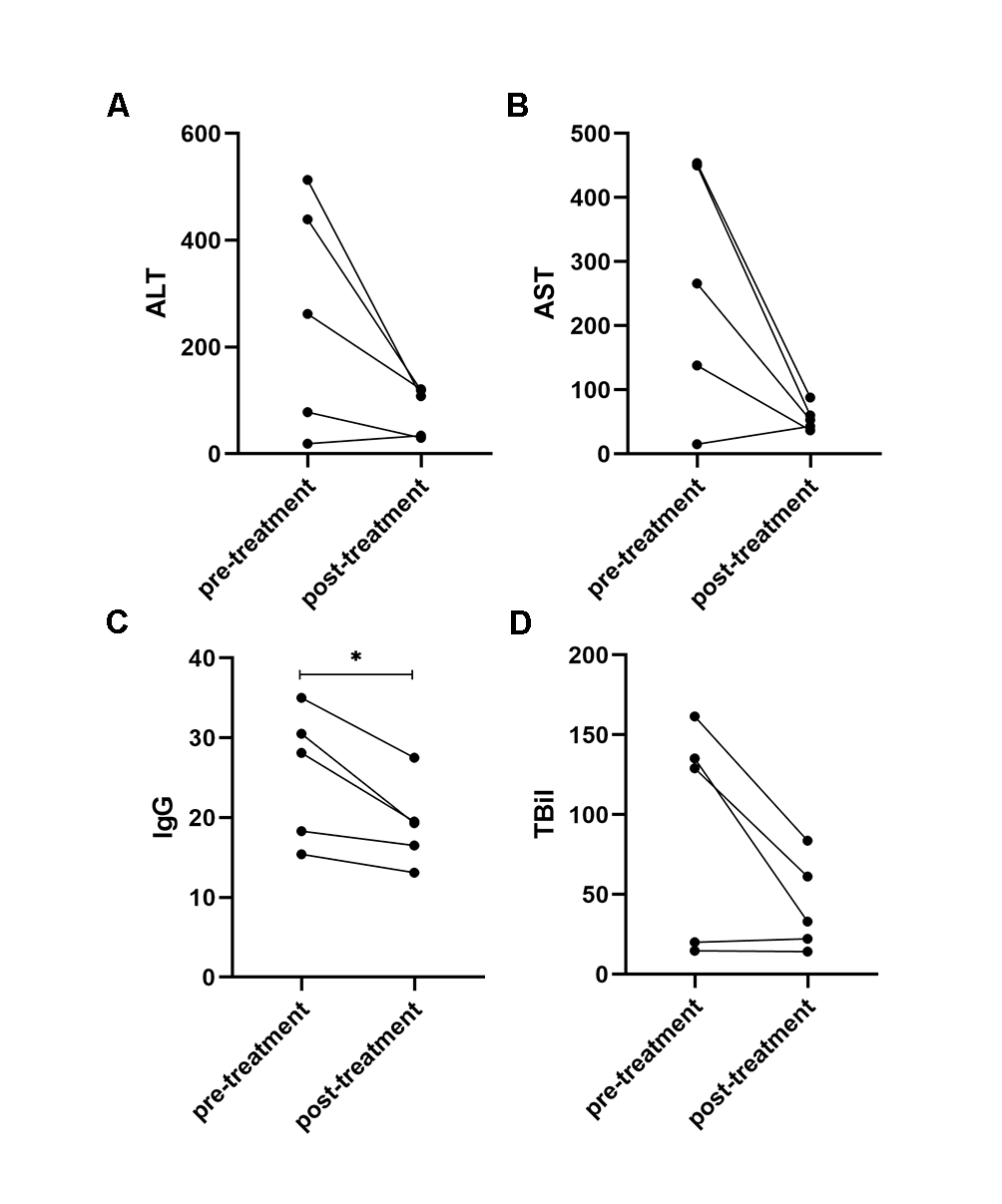

Supplement: Supplementary file 4 [file Image4.TIF]

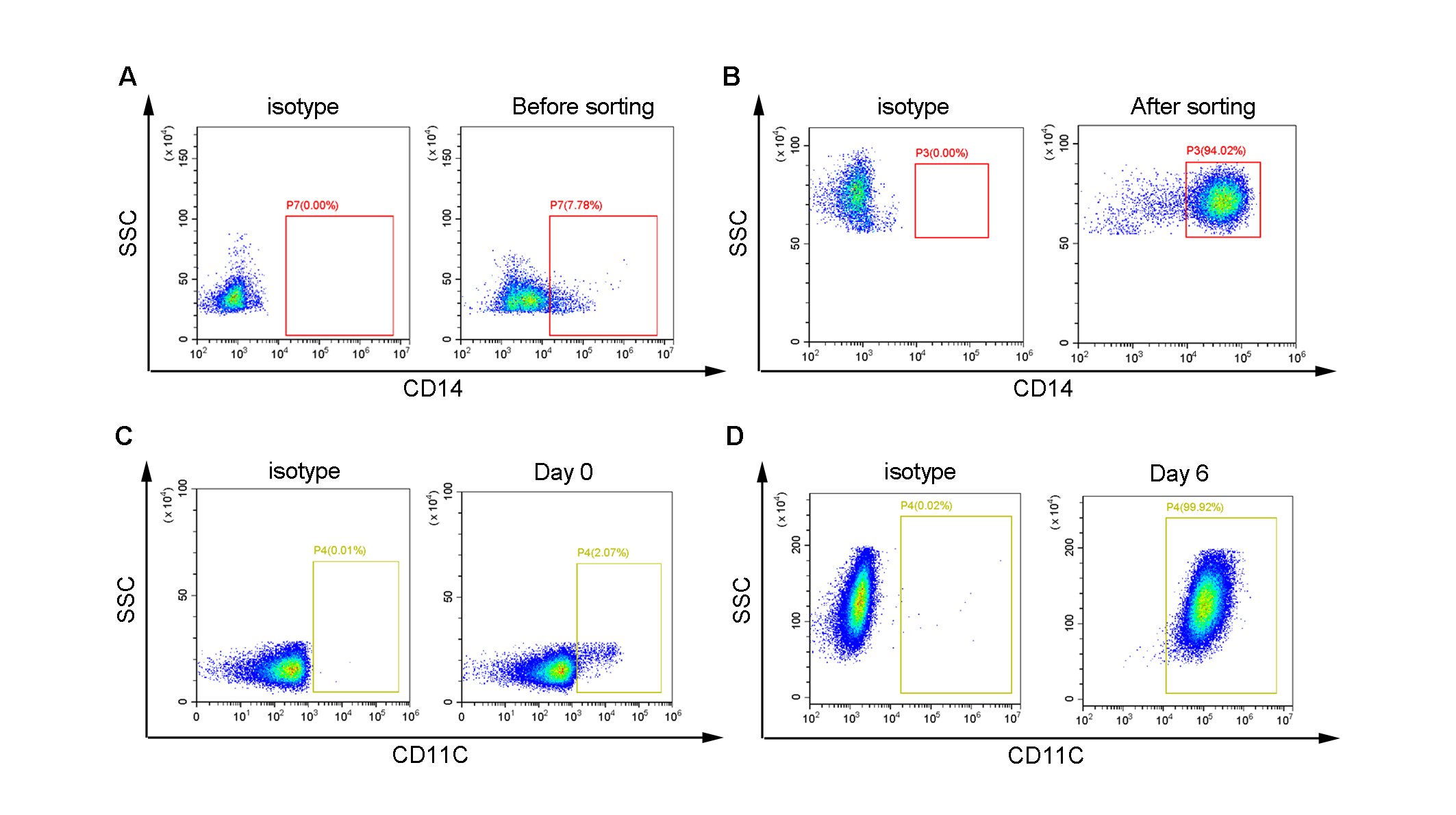

Supplement: Supplementary file 5 [file Image2.TIF]

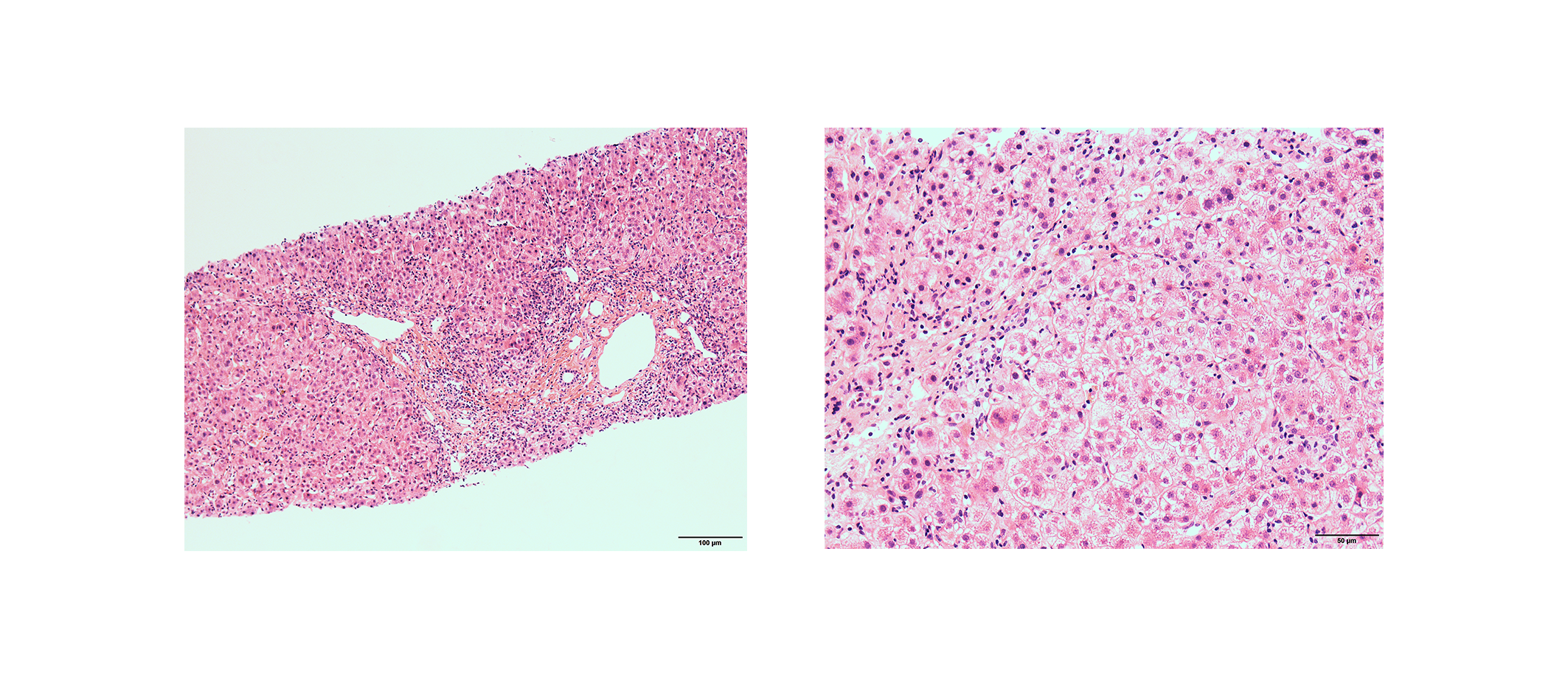

Supplement: Supplementary file 6 [file Image1.TIF]

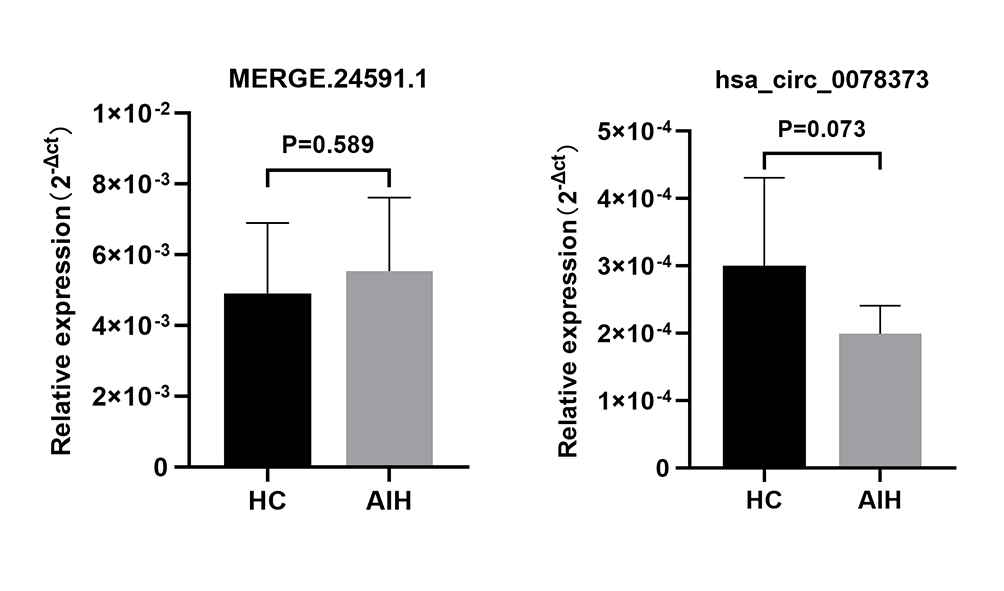

Supplement: Supplementary file 10 [file Image5.TIF]
