# Supplementary material for: Long Noncoding RNA and Circular RNA Expression Profiles of Monocyte-Derived Dendritic Cells in Autoimmune Hepatitis
Source: Front Pharmacol. 2021 Dec 6;12:792138. doi: 10.3389/fphar.2021.792138 (PMC8685411; doi:10.3389/fphar.2021.792138)
Supplement: Supplementary file 7 [file DataSheet1.PDF]

Supplementary Table 1. Histological features of patients with AIH

**Histological features**	**Amount (n=44)**
Interface inflammation	44/44 (100%)
Hepatocyte rosettes	35/44 (79.5%)
Periportal lymphocyte	44/44 (100%)
Simplified IAIHG score	
≥ 6	5/44 (11.4%)
≥ 7	39/44 (88.6%)
G (0/1/2/3/4)	0/0/13/30/1
S (0/1/2/3/4)	0/5/16/15/8

IAIHG, international autoimmune hepatitis group score; G, the grade of inflammation; S, the stage of fibrosis.

Supplementary Table 2. Clinical characteristics of patients with AIH

**Clinical characteristics**	**Amount (n=27)**
Age, years	50.1 ±12.1
Female	25/27 (92.6%)
Liver function indexes	
TBil, µmol/L	92.4 ± 95.00
ALT, IU/L	233.7 ± 205.7
AST, IU/L	340.6 ± 430.4
Immunoglobulin	
IgG, IU/L	26.7 ± 13.9
ANA (+, N%)	26/27 (96.3%)

TBil, total bilirubin; ALT, alanine aminotransferase; AST, aspartate aminotransferase; IgG, Immunoglobulin G; ANA, antinuclear antibody.

Supplementary Table 3. Clinical characteristics of 5 AIH patients before treatment.

Clinical characteristics	Amount (n=5)
Age, years	58.3 ±11.1
Female	4/5 (80%)
Liver function indexes	
TBil, μmol/L	92.1 ± 62.0
ALT, IU/L	262.2 ± 193.5
AST, IU/L	264.6 ± 172.4
Immunoglobulin	
IgG, IU/L	25.5 ± 7.4
ANA (+, N%)	4/5 (80%)

TBil, total bilirubin; ALT, alanine aminotransferase; AST, aspartate aminotransferase; IgG, immunoglobulin G; ANA, antinuclear antibody.

Supplementary Table 4. Clinical characteristics of AIH patients used for qPCR

Clinical characteristics	Amount (n=17)
Age, years	55.4 + 7.9
Female	16/17 (94.1%)
Liver function indexes	
TBil, μmol/L	75.3 ± 78.4
ALT, IU/L	162.0 ± 108.7
AST, IU/L	275.6 ± 205.5
Immunoglobulin	
IgG, IU/L	28.2 ± 10.0
ANA (+, N%)	16/17 (94.1%)

TBil: total bilirubin, ALT: alanine aminotransferase, AST: aspartate aminotransferase, IgG: immunoglobulin G, ANA: antinuclear antibody.

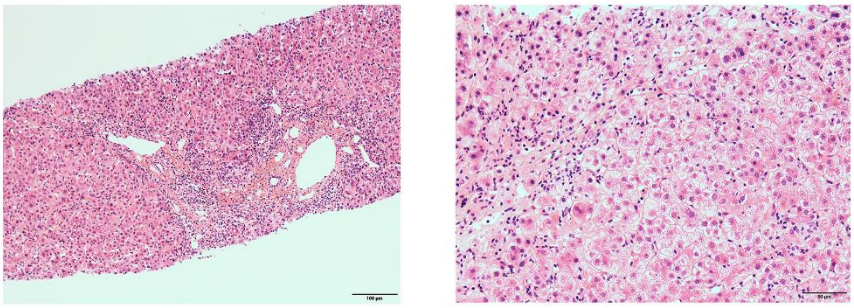

Supplementary Figure 1: Histological features of patients with AIH

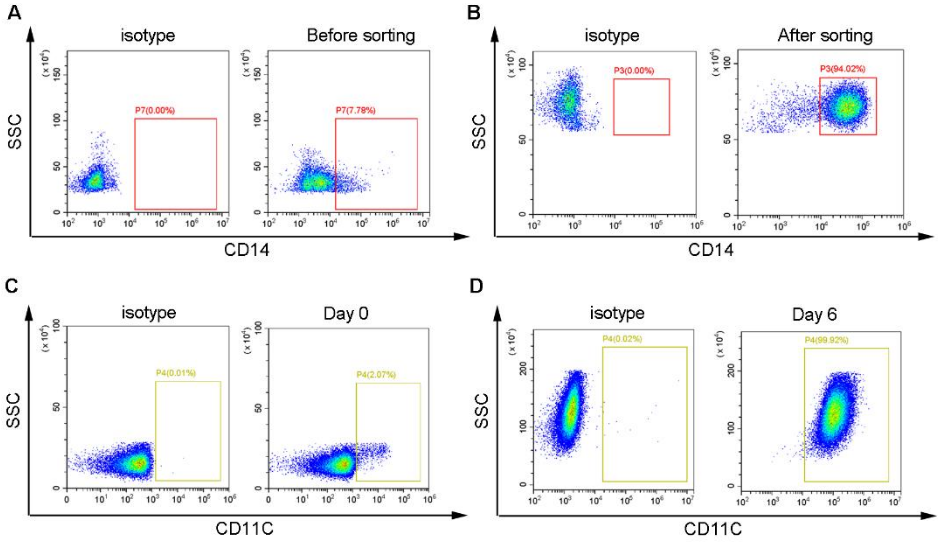

Supplementary Figure 2: (A and B) After sorting with CD14$^+$ magnetic beads, the proportion of CD14$^+$ cells was as high as 94%. (C and D) The percentage of CD11c$^+$ DCs significantly increased from 2.07% to 99%. The induced culture of moDCs was successful.

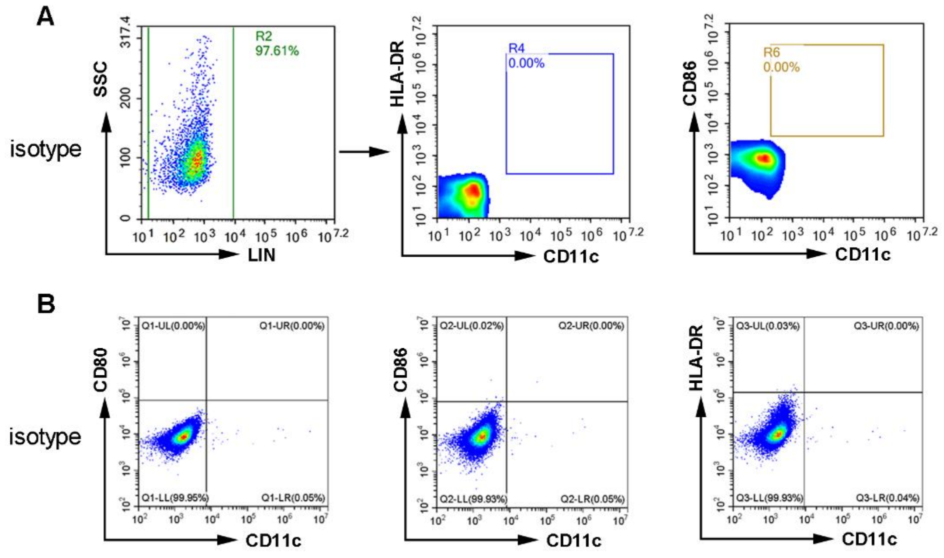

Supplementary Figure 3: (A) the antibody isotypes of lineage, CD11c, HLA-DR, CD86 used in the flow cytometric analysis of PBMCs from AIH patients and healthy controls. (B) the antibody isotypes of CD11c, HLA-DR, CD86, CD80 used in flow cytometric analysis of moDCs from AIH patients and healthy controls.

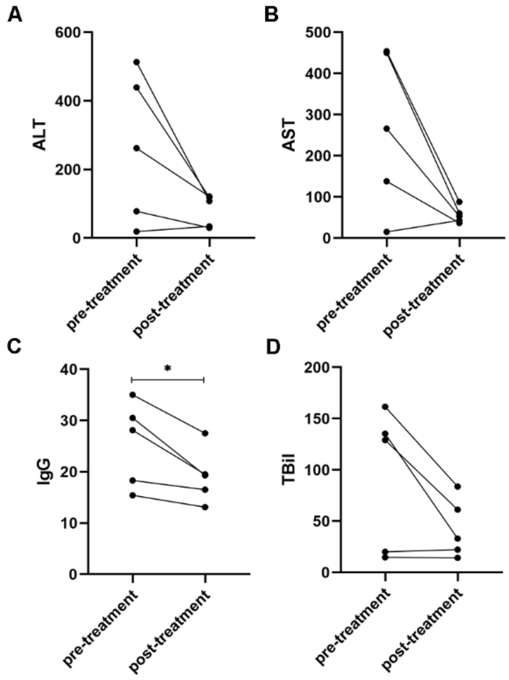

Supplementary Figure 4: Changes of laboratory indexes in AIH patients before and after 2-3 weeks of oral MP treatment. ALT, alanine aminotransferase; AST, aspartate aminotransferase; IgG, immunoglobulin G; TBil, total bilirubin. *$p < 0.05$

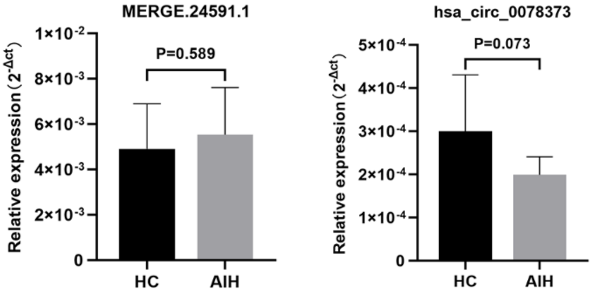

Supplementary Figure 5: Validation of lncRNA MERGE.24591.1 and has_circ_0078373 in moDCs from healthy controls and AIH patients.
